# Catan-ionic hybrid lipidic nano-carriers for enhanced bioavailability and anti-tumor efficacy of chemodrugs

**DOI:** 10.18632/oncotarget.15942

**Published:** 2017-03-06

**Authors:** Bilin Liu, Dan He, Jianyong Wu, Quan Sun, Mi Zhang, Qunyou Tan, Yao Li, Jingqing Zhang

**Affiliations:** ^1^ Chongqing Research Center for Pharmaceutical Engineering, Chongqing Medical University, Chongqing 400016, China; ^2^ Department of Thoracic Surgery, Institute of Surgery Research, Daping Hospital, Third Military Medical University, Chongqing 400016, China

**Keywords:** catan-ionic nano-vesicles, hybrid lipidic nano-carries, effective delivery, bioavailability, antitumor efficacy

## Abstract

To date there has not been any report on catan-ionic hybrid lipidic nano-carriers, let alone a report on applying them to deliver insoluble anti-tumor drugs. Catan-ionic hybrid lipidic nano-carriers containing curcumin (CUR-C-HLN) inherit the merits of catan-ionic systems, hybrid lipidic systems and nano-structured carriers (the second-generation substitute of solid lipidic nano-systems). Catan-ionic surfactants increased microvesicle stabilization by producing unordered isometric clusters, enhanced absorptive amount as an inhibitor of enzyme and protein, improved tumor accumulation by cellular endocytosis and membranous fusion; hybrid lipids helped to obtain high drug content and low leakage by forming a less-organized matrix arrangement. CUR-C-HLN favorably changed absorptive and pharmacokinetic properties after oral and/or intravenous administrations; improved cell growth inhibition, apoptotic inducing and anti-invasion effects; enhanced antitumor efficiency and reduced cancerous growth. Catan-ionic hybrid lipidic nano-carriers provide an alternative good choice for effective delivery of anticancerous chemodrugs.

## INTRODUCTION

The most frequently used chemotherapy drugs for non-small cell lung cancer (NSCLC) [1], such as cisplatin and vinblastine, have high anti-tumor activity and severe toxic effects [2, 3]. It is necessary to create novel, efficacious and harmless treatment methods. Curcumin (CUR), a polyphenol extracted from the herb turmeric, shows cytotoxicity in three main kinds of NSCLCs: adenocarcinoma [4], squamous [5] and large-cell cancers [6]. Furthermore, CUR shows no effects on normal cells or animal models tested at a high dose of up to 10 g per day [7]. But, like other chemodrugs, further application of CUR has been seriously obstructed by unsatisfactory pharmcokinetic properties and poor tumor cell targeting.

Lipidic nano-systems [8, 9], including the early emerging systems (nano-emulsions and nano-liposomes) [10–12] and the later emerging ones (solid lipidic nano-particles) [13], are already used in clinical practice. Hybrid lipidic nano-carriers (HLNs), being second-generation substitute of solid lipidic nano-particles, have been developed in the last decade and able to compensate the drawbacks of solid lipidic nano-particles. HLNs have a higher drug loading capacity and element drug stacking [14] and less incorporation of drug expulsion from the vesicles compared to first-generation nano-systems because HLNs conceive a less-organized matrix arrangement by blending fluid lipids with solid lipids [15]. HLNs have shown the potential for drug deliveries, such as gene delivery [16], chemical drug delivery [17] and herbal drug delivery [18]. In the case of antitumor drugs, HLNs have been investigated for nasal delivery of CUR to increase its cytotoxicity against astrocytoma-glioblastoma cells [19], lung delivery of anticancerous chemicals and siRNA to suppress lung tumor growth and prevent adverse side effects [20], intravenous delivery of tributyrin to improve anti-breast cancer activity [21], oral delivery of tamoxifen to enhance *in vivo* antitumor efficacy with reduced adverse drug effects [22], etc. The catan-ionic nano-systems consist of positive and negative charged surfactants. They have raised great interests lately, since they possess increased stability [23], absorptive amount [10, 24] and cellular accumulation [25]. It is reasonable to assume that the catan-ionic HLNs (C-HLN) inherit the merits of HLNs and catan-ionic systems and act as a new generation of versatile therapeutic delivery platforms. Obviously, laboratory experiments and animal trials are crucial to judge this hypothesis. However, to date there has not been any report on C-HLN in general or on applying C-HLN to deliver insoluble chemodrugs.

Herein, catan-ionic hybrid lipidic nano-carriers containing CUR (CUR-C-HLN) are design and fabricated to effectively deliver CUR for the first time. Compared with the catan-ionic solid lipidic nano-systems [23] recently reported by us, CUR-C-HLN might be served as the second generation catan-ionic lipidic nano-systems integrating the merits of catan-ionic systems, hybrid lipidic systems and nano-structured carriers (the second-generation of lipidic nano-systems). CUR-C-HLN significantly improves bioavailability and greatly increases anti-lung cancerous effects of CUR. CUR-C-HLN was fabricated according to the optimized formula and characterized by Fourier-transformed infrared (FTIR) and differential scanning calorimetry (DSC). The features of CUR-C-HLN as nano-carriers of chemodrugs were investigated according to micro-morphology, drug content, encapsulation ratio, release amount, and *in vitro* cytotoxicity. Additionally, the bioavailability and pharmacokinetic characteristics were evaluated via two administration routes. The antitumor efficacies in cancerous mice were evaluated.

## RESULTS

### Fabrication of CUR-C-HLN

**Figure 1:**
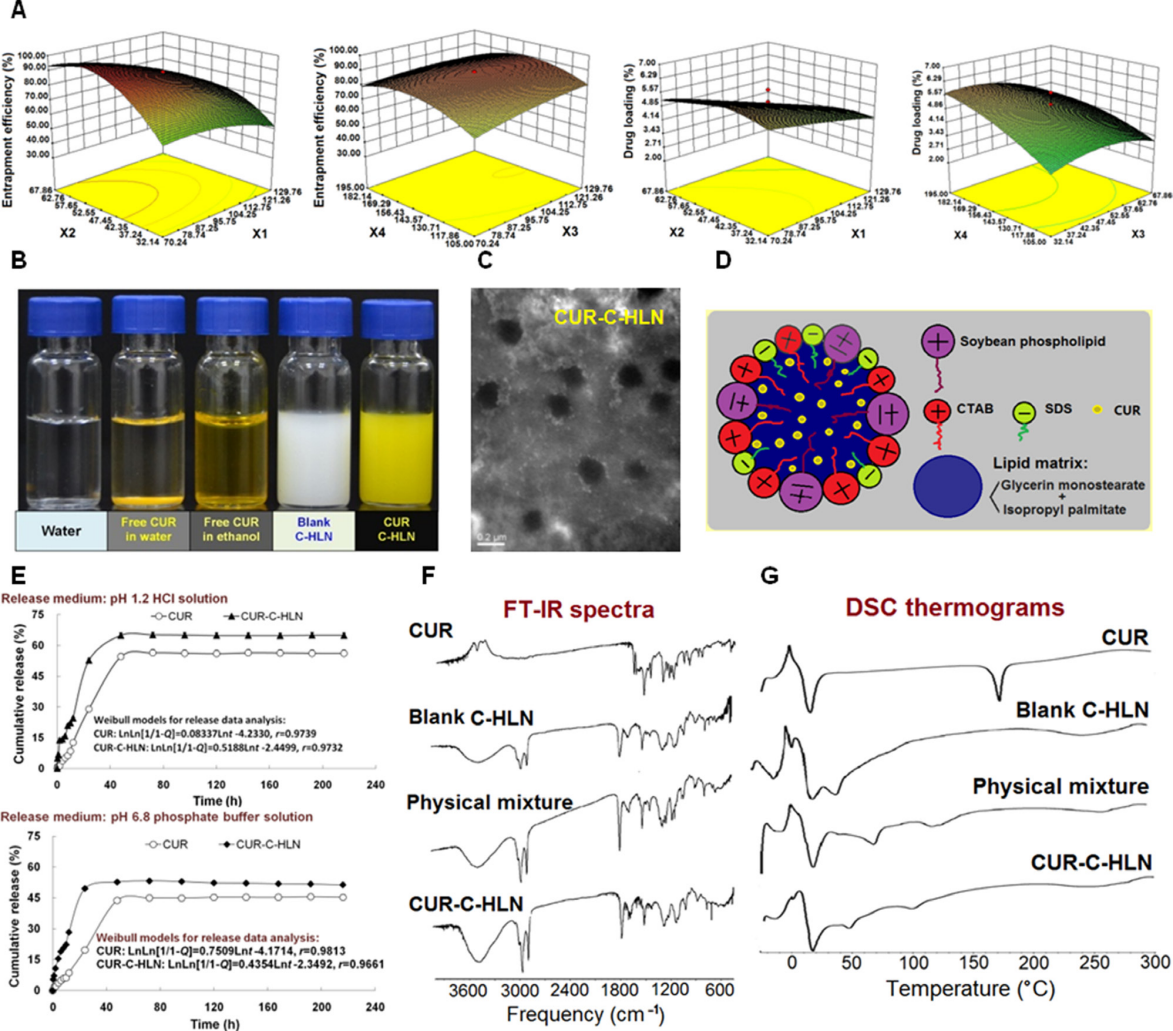
Preparation and elementary characteristics of CUR-C-HLN (**A**) Formulation optimization (mean ± SD, *n* = 3). (**B**) optical photographs. (**C**) transmission electron photomicrographs (bar: 200 nm). (**D**) schematic illustration of the CUR-C-HLN structure. (**E**) *in vitro* release behaviors (mean ± SD, *n* = 3), (**F**) FT-IR spectra and (**G**) DSC thermograms of CUR-C-HLN.

The CUR-C-HLNs were formulated and fabricated. The CUR-C-HLNs, with a size of 235.9 ± 9.6 nm, were evenly dispersed. The encapsulation ratio and drug content were 93.23 ± 1.23 % and 6.75 ± 2.62 % (*n* = 3), respectively (Figure 1A). The yellow CUR-C-HLN suspensions (Figure 1B) tended to be circular and dispersed separately (Figure 1C). Their electric potentials were negative (–28.40 ± 0.35 mV, *n* = 3), which might help keep these nano-particles stable [26]. The pH and conductivities of CUR-C-HLN were 5.83 ± 0.01 and 613.33 ± 3.21 μs/cm (*n* = 3, 25°C), respectively (Supplementary Table 1). As shown in Figure 1D, CUR-C-HLNs basically consisted of a binary lipid matrix of solid lipidic (glycerin monostearate) and liquid lipidic substances (isopropyl palmitate), the zwitter-ionic surfactant and emulsifying agent (phospholipid), positive-charged surfactant (glycerin monostearate, cetyltrimethyl ammonium bromide, CTAB) and negative-charged surfactant (sodium dodecyl sulfate, SDS). The above two surfactants comprised the catan-ionic surfactants.

The optimal formula was obtained by a four-factor and five-level central composite design-response surface methodology (CCD-RSM) (Figure 1A). The encapsulation ratio (between ~12% and ~96%) and drug content (between ~1% and ~7%) of 30 lots changed significantly (Supplementary Table 2). The fitting models are listed as follows:

$$\begin{array}{l} Y_{1}\;=\;88.90\;-12.09\;X_{1}+\;8.23\;X_{2}+3.15\;X_{3}\;+\;2.60\\ X_{4}\;-\;5.89\;X_{1}\;X_{2}\;+\;5.66\;X_{1}\;X_{3}\;+\;1.36\;X_{1}\;X_{4}\;+\;4.90\\ X_{2}\;X_{3}-4.42\;X_{2}\;X_{4}-2.16\;X_{3}\;X_{4}-5.29\;X_{1}^{2}-16.71\\ X_{2}^{2}-32.01\;X_{3}^{2}-9.18\;X_{4}^{2}\;\left(r=0.9739\right) \end{array}$$ (1)

$$\begin{array}{l} Y_{2}\;=\;5.05\;-\;0.84\;X_{1}-0.39\;X_{2}\;+\;0.70\;X_{3}\;+\;0.86\\ X_{4}-0.31\;X_{1}\;X_{2}-0.36\;X_{1}\;X_{3}-0.49\;X_{1}\;X_{4}\;+\;0.48\\ X_{2}\;X_{3}-0.28\;X_{2}\;X_{4}-0.29\;X_{3}\;X_{4}-0.057\;X_{1}^{2}-0.54\\ X_{2}^{2}-0.33\;X_{3}^{2}-0.34\;X_{4}^{2}\;\left(r=0.9543\right) \end{array}$$ (2)

As shown in the above equations and past research [27, 28], the optimal values for *X*_1_, *X*_2_, *X*_3_ and *X*_4_ should be 70.24 mg, 54.07 mg, 117.35 mg and 168.77 μmol, respectively. The predicted values and the experimental results of CUR-C-HLN prepared under the optimal protocol were fairly consistent (Supplementary Table 3).

### Elementary properties of CUR-C-HLN

As depicted in Figure 1E, CUR-C-HLN could obviously increase the *in vitro* release amounts of CUR in the simulated human digestive fluids. The Weibull models were used for fitting the release data (Table 1). Statistically differences existed between release data of CUR-C-HLN and CUR (Supplementary Table 4). As shown in Figure 1F, the peaks or the intensities of 1508 and 1604 cm^−1^ (benzene ring: stretching vibration), 1629 (unsaturated ketone: stretching vibration) in the IR spectro-scopy of the physically mixing compound reduced or disappeared in the CUR-C-HLN curve. As depicted in Figure 1G, the calorimetric curve of CUR presented a peak at 177°C (melting point). The endothermic peaks of 20°C, 70°C and 121°C in the curve of the mixing compound changed to peaks of 17°C, 47°C and 99°C with lower intensities in the CUR-C-HLN spectrum, which suggested that CUR had been enclosed in the C-HLNs. It was noted that the peak at 177°C of CUR in the thermogram disappeared in the physical mixture, which might be explained by the theory of preparation by melt-out method [27]; i.e., when the temperature rose, C-HLN melt and CUR dissolved in the C-HLN, partially forming the complex.

**Table 1:** Mathematical models of mean cumulative release rate versus time of CUR-C-HLN and free CUR

Formulation	Release medium	0.1 mol/L HCl	pH 6.8 PBS
CUR-C-HLN	Zero-order kinetic model	*Q* = 0.2887*t* + 20.612, *r* = 0.8114	*Q* = 0.2099*t* + 20.072, *r* = 0.7680
	First-order kinetic model	ln(1-*Q*)=–0.0051*t*-0.2667, *r* = 0.8319	ln(1-*Q*) = –0.0032*t*-0.2480, *r* = 0.7799
	Higuchi model	*Q* = 4.8095*t*^1/2^ + 8.8444, *r* = 0.9187	*Q* = 3.5860*t*^1/2^ + 10.9610, *r* = 0.8888
	Hixcon-Crowell model	(100-*Q*)^1/3^ = –0.0064t + 4.2657, *r* = 0.8267	(100-*Q*)^1/3^= –0.0043*t* + 4.2855, *r* = 0.7767
	Ritger-peppas model	ln*Q* = 0.4254ln*t* + 2.1506, *r* = 0.9721	ln*Q* = 0.4589ln*t* + 1.9348, *r* = 0.9720
	Weibull model	lnln[1/1-*Q*] = 0.5188ln*t*-2.4499, *r* = 0.9732	lnln[1/1-*Q*] = 0.4354ln*t*-2.3492, *r* = 0.9661
CUR	Zero-order kinetic model	*Q* = 0.2969*t* + 10.156, *r* = 0.8545	*Q* = 0.2400*t* + 8.0380, *r* = 0.8626
	First-order kinetic model	ln(1-*Q*) = –0.0045*t*-0.1262, *r* = 0.8626	ln(1-*Q*) = –0.0033*t*-0.0936, *r* = 0.8678
	Higuchi model	*Q* = 4.8079*t*^1/2^-1.08825, *r* = 0.9369	*Q* = 3.8636*t*^1/2^-0.9135, *r* = 0.9405
	Hixcon-Crowell model	(100-*Q*)^1/3^ = –0.0060*t* + 4.4609, *r* = 0.8602	(100-*Q*)^1/3^ = –0.0046t + 4.5044, *r* = 0.8661
	Ritger-peppas model	ln*Q* = 0.7515ln*t* + 0.04172, *r* = 0.9725	ln*Q* = 0.6903ln*t* + 0.4649, *r* = 0.9820
	Weibull model	lnln[1/1-*Q*] = 0.0834ln*t*-4.2330, *r* = 0.9739	lnln[1/1-*Q*] = 0.8337ln*t*-4.2330, *r* = 0.9739

*Q* = cumulative CUR release at time *t*.

### Perfusion of CUR-C-HLN to improve the absorptive behavior

**Figure 2:**
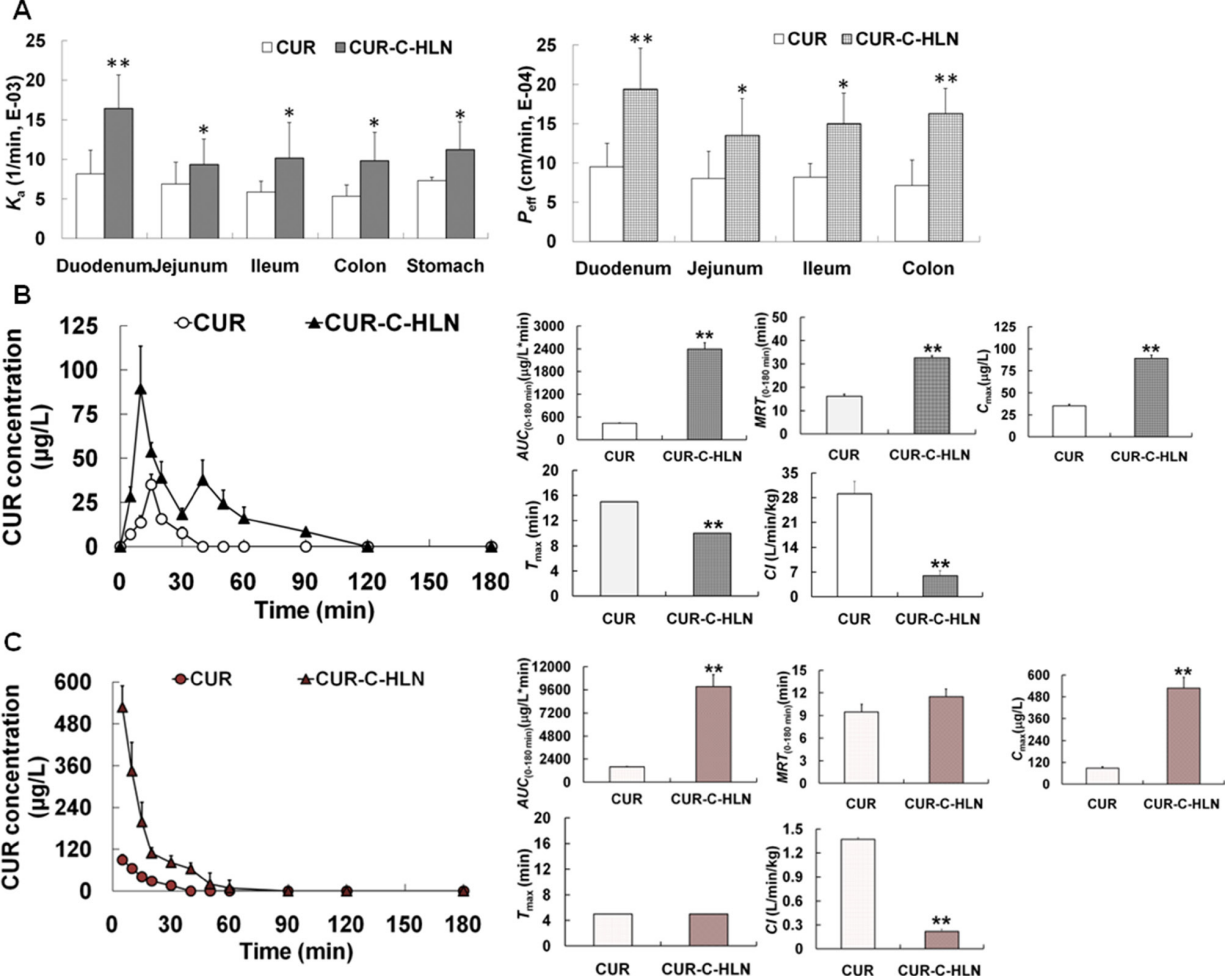
*In situ* absorptive and *in vivo* kinetic characteristics of CUR-C-HLN (**A**) The absorption rate constant (*K*_a_) and effective permeability (*P*_eff_) of CUR-C-HLN. (**B**) Plasma CUR concentration versus time profiles and pharmacokinetic parameters of CUR after oral administration at the dose of 15 mg/kg. (**C**) Plasma CUR concentration versus time profiles and pharmacokinetic parameters after intravenous injection at the dose of 2.5 mg/kg. The data are shown as the mean ± SD. *n* = 6 rats per group. *P < 0.05 or **P < 0.01 indicate significant differences between CUR-C-HLN and CUR.

The duodenum was the major site for CUR absorption, which were markedly enhanced by entrapping CUR inside the C-HLN vesicles (Figure 2A). CUR-C-HLN also enhanced the absorptive amount of CUR in other gastro-intestinal segments. The absorptive rate and permeability of CUR-C-HLNs in the stomach, duodenum, colon, ileum and jejunum were separately 1.54-fold, 2.01-fold (or 2.04-fold), 1.85-fold (or 2.29-fold), 1.74-fold (or 1.84-fold) and 1.35-fold (or 1.69-fold) those of CUR.

### Improved pharmacokinetic properties of CUR-C-HLN via oral administration

As shown in Figure 2B, the pharmacokinetic behavior of CUR-C-HLN was more desirable compared to free CUR. The CUR contents of CUR-C-HLN were much larger than those of CUR. The maximal contents came up at 10 min and 40 min for CUR-C-HLN or 15 min for CUR, respectively.

Compared to free CUR, CUR-C-HLN possessed favorable pharmacokinetic behavior. For example, the values of CUR-C-HLN, such as the area under concentration versus time profile (*AUC*), maximal concentration (*C*_max_) and mean residence time (*MRT*) were 5.51-fold, 2.54-fold, 2.03-fold higher while the clearance rate (*Cl*) were much slower (only one fifth). The relative bioavailability of CUR-C-HLN versus CUR was 551.08%. Furthermore, by computating the confident levels of *AUC* and *C*_max_, and testing the *T*_max_ values using the Wilcoxon rank sum (Table 1), bioequivalence of CUR-C-HLN and CUR was considered not to be established.

### Improved pharmacokinetic properties of CUR-C-HLN via intravenous administration

After the rats were intravenously given with CUR-C-HLN and free CUR, their pharmacokinetic behaviors were presented in Figure 2C. The CUR concentrations of CUR-C-HLNs decreased sharply in the first ten min (from ~530 ng/mL at 5 min to ~200 ng/mL at 15 min) and decreased slowly for the following 75 min (undetectable in 90 min). By comparing the *AUC* values, the bioavailability of CUR-C-HLN to CUR was 618.20%. After intravenous injection, bioequivalence of CUR-C-HLN and CUR was considered not to be established (Table 2), and it had higher *AUC*, *MRT*, *C*_max_ and lower *Cl* values (6.18, 1.21, 5.91 and 0.16 times that of CUR).

**Table 2:** Bioequivalence evaluations of CUR-C-HLN and CUR after oral administration at the CUR dose of 15 mg/kg or intravenous injection at the CUR dose of 2.5 mg/kg

Administration route	Parameter	90% confidence interval calculated	*P* value calculated	Bioequivalence standard	Bioequivalence
Oral administration	*AUC*	464.88%–633.13%	-	80%–125%	No
	*C*_max_	189.19%–319.46%	-	70%–143%	No
	*T*_max_	-	< 0.05	> 0.05	No
	In all	-	-	-	No
Intravenous injection	*AUC*	565.32%–671.09%	-	80%–125%	No
	*C*_max_	546.04%–636.78%	-	70%–143%	Yes
	*T*_max_	-	< 0.05	> 0.05	No
	In all	-	-	-	No

Note: Data presented as the mean ± standard deviation (*n* = 6).

**Figure 3:**
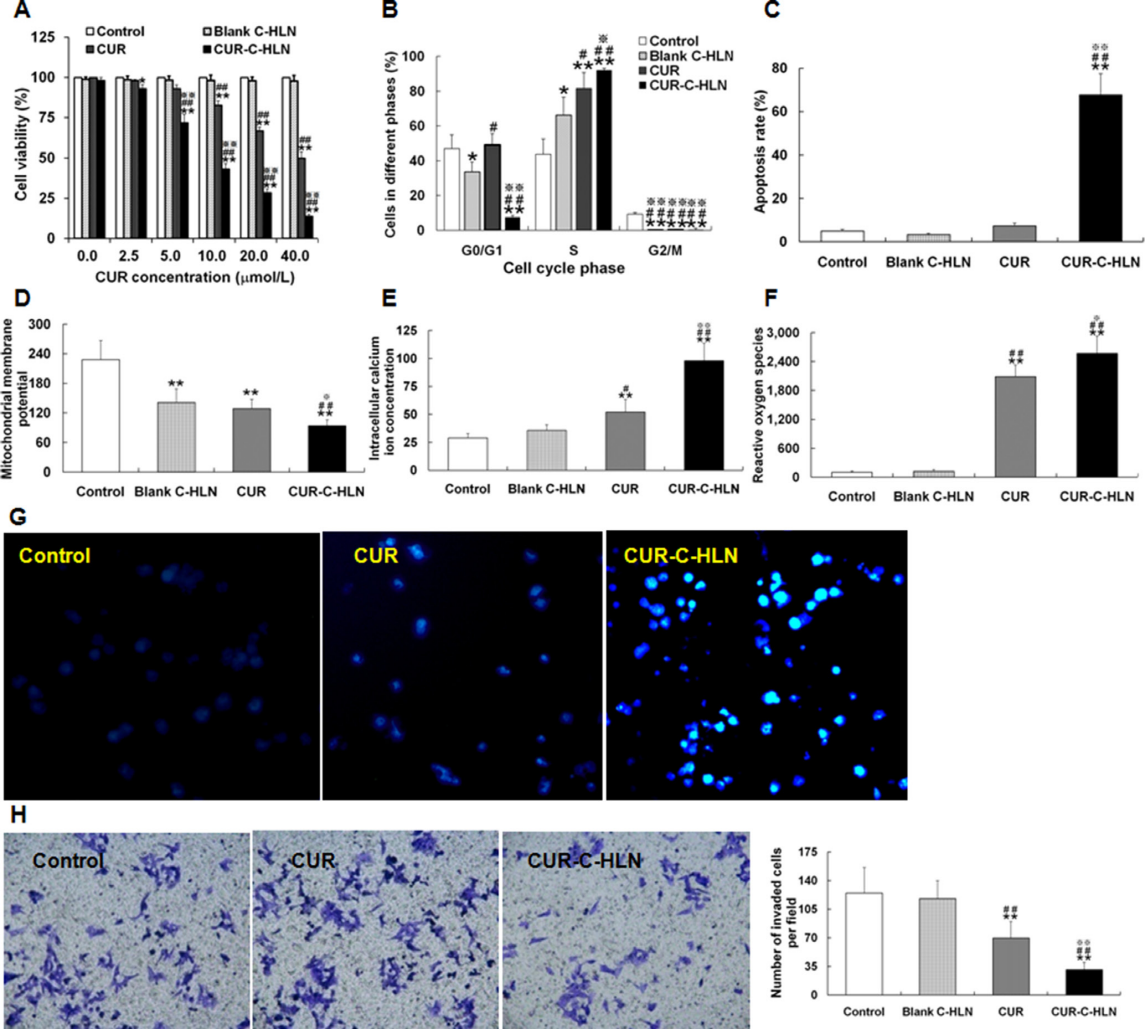
Effects of CUR-C-HLN on Lewis lung cancer cells (**A**) Cell viability phase after 24 h’ exposure to different concentrations of CUR-C-HLN and free CUR. (**B**) cell cycle, (**C**) apoptosis rate, (**D**) mitochondrial membrane potential, (**E**) intracellular calcium ion levels and (**F**) reactive oxygen species of LLC cells after 24 h’ exposure to 20 μmol/L CUR-C-HLN and free CUR. (**G**) The inverted photomicrographs of LLC cells after 24 h’ exposure to 5 μmol/L CUR-C-HLN and free CUR. (**H**) Representative cellular field images of Matrigel-invaded LLC cells at 400× magnification, and bar graphs representing the average invaded cells per field. The results were presented as the mean ± SD (*n* = 3), *P < 0.05 for the test sample compared with negative control, ^#^*P* < 0.05 for the test sample compared with Blank C-HLN, *P* < 0.05 for the test sample compared with free CUR.

CUR-C-HLN and free CUR inhibited the cancerous cell growth in a dose dependent way (Figure 3A). The 50% inhibitive concentrations of CUR-C-HLN and CUR at 24 h were 8.81 and 39.70 μmol/L, respectively. The quantity of cells dealt with CUR-C-HLN were arrested in the S phase of cell cycle (~92%) much more than that with CUR (~82%), C-HLN (~66%) and the control (~41%) (Figure 3B). Similarly, as shown in Figure 3C, the CUR-C-HLN-inducing apoptosis ratio (~68%) was much higher than that of CUR (~7%). The apoptosis induction of C-HLN and the negative control in cancerous cells were ignorable.

The membranous potential of mitochondria in LLC cells dealt with 20 μmol/L CUR-C-HLN and CUR decreased a lot than that without treatment (the negative control), but these two were significantly different (Figure 3D). The calcium ion content measured in tumor cells treated with CUR-C-HLN was 2-fold that of CUR and 3-fold that of C-HLN and the control (Figure 3E). Similarly, the active oxygen content in cells dealt with CUR-C-HLN was much higher than that without treatment or dealt with C-HLN (Figure 3F). As presented in Figure 3G, the nuclei was appropriately stained and the characteristics of apoptosis was readily visible in cells dealt with CUR-C-HLNs and CUR for 1 d.

The anti-invasion effects of CUR-C-HLN were observed by Transwell method. The invasive capabilities of the tumor cells without treatment were 4.0 times and 1.8 times that of the cells dealt with CUR-C-HLN and CUR (Figure 3H), respectively.

### Antitumor efficiency of CUR-C-HLN

**Figure 4:**
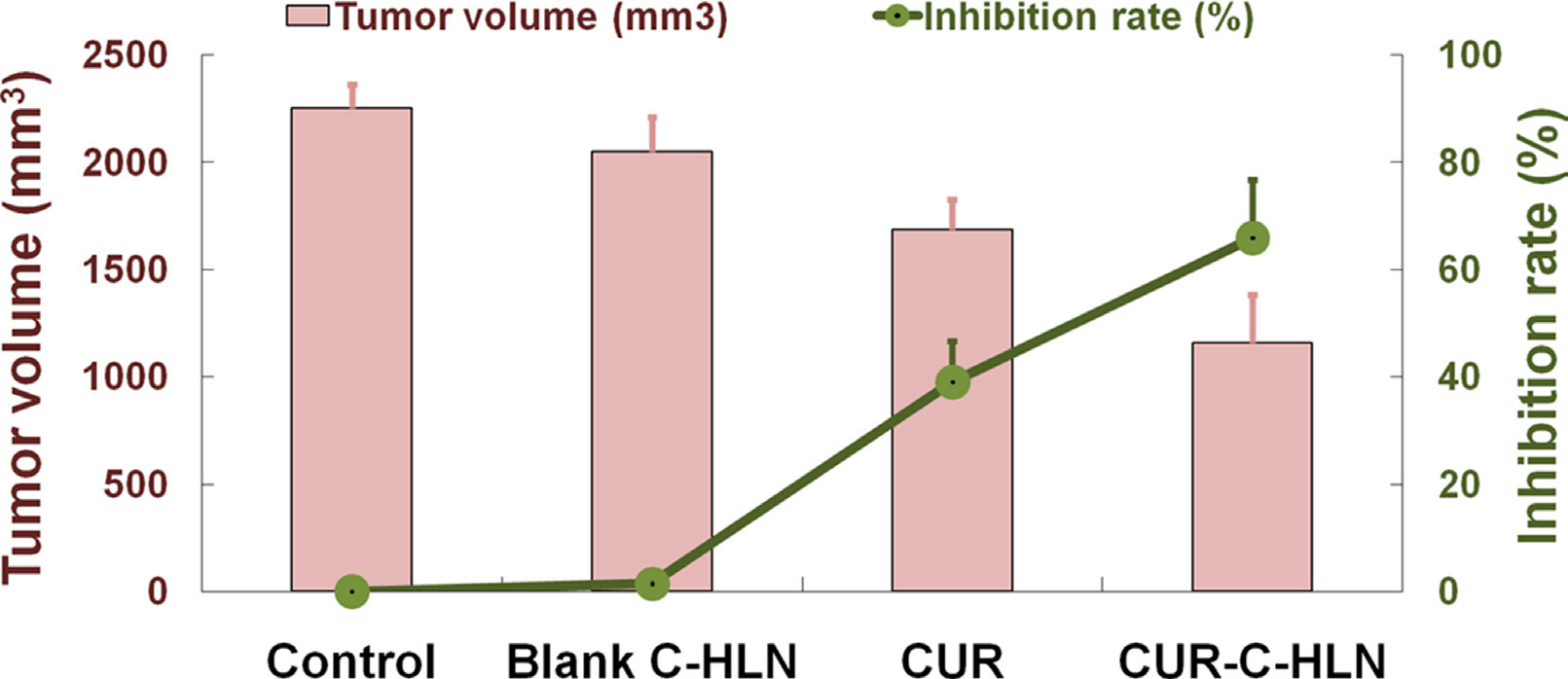
Effects of CUR-C-HLN on tumor volumes and weights in LLC cells-bearing mice The data are shown as the mean ± SD (*n* = 6).

As shown in the Figure 4, the tumor volumes and weights of cancerous mice administered with CUR-C-HLN had the slowest increasing rate, compared with that of mice in other three groups. The tumor inhibitory rate (~70%) in mice given with CUR-C-HLN was the highest, while the rate (~40%) in mice given with free CUR was much lower.

## DISCUSSION

According to a report from World Health Organization, the mortality and incidence of lung cancer in 2014 were unexpectedly high [29]. Chemotherapy is an effective method for inoperatable patients or patients with more advanced cancer, or is applied before surgery as a neoadjuvant therapy or after surgery as an adjuvant therapy [1]. CUR continues to attract more and more attention owing to its high safety and anticancerous activities against many cancerous cells [30]. In the last several decades, nanotechnologies have been introduced to overcome the application limitations of CUR such as low bioavailability and anticancerous effects. For examples, PLGA nano-particles [31] and diblock copolymer micelles [32] were fabricated to improve the anti-breast cancer activity of CUR; lipidic nano-particles containing CUR and doxorubicin were prepared to enhance the treatment effects of hepatocellular carcinoma [33]; and polyethylene glycol derivatives of vitamin E succinate self-assembled nano-particles containing CUR and sorafenib were prepared to enhance the curative effects on hepatocellular carcinoma [34].

Here, we first reported on a class of nanocarriers, i.e., catan-ionic hybrid lipidic nano-particles. These nano-carriers could efficiently deliver CUR to achieve enhancement of bioavailability via oral and intravenous routes and improve anti-lung cancerous effects. Unlike common lipidic nano-particles, CUR-C-HLNs had catan-ionic surfactants and binary lipids (Figure 1D). The positive and negative charged surfactants (catan-ionic surfactants), the zwitter-ionic surfactant and emulsifying agent existed in the interfacial region of nano-carriers and the external water by means of self-shaping into a stable shell that surrounded the nano-particles. The catan-ionic surfactants were also able to enhance CUR accumulation in cancerous cells following endocytosis or membranous fusion [25]. Hybrid lipidic substances (a blended lipidic matrix of a solid lipidic glycerin monostearate and a fluid lipidic isopropyl palmitate) made up the core of the nano-particles, held the insoluble CUR as much as possible, and avoided drug expulsion by conceiving a less-organized matrix alignment [35]. The preparation process of CUR-C-HLN was easily manipulated and suitable for large-scale production.

The optimal recipe for CUR-C-HLN was obtained using a four-factor and five-level CCD-RSM method [27, 28]. Our tests helped to understand the complex relationship between the variables (vesicular components) and the response (CUR content and encapsulation ratio). The encapsulation ratio of CUR-C-HLN was higher than that of the other core-shell delivery systems (such as mPEG-PCL micelles) [36] or lung-targeted delivery systems (such as gelatin microspheres) (88 ± 3.32% or 75.5 ± 3.82%) [37]. The infrared and calorimetric data of CUR-C-HLN suggested that CUR was successfully entrapped in the nano-particles.

Compared to free CUR, CUR-C-HLN obviously enhanced the *in situ* absorptive rate and permeability of CUR which undoubtedly contribute to the gastro-intestinal absorptive amount. However, although it was reported that the concentration-independent intestinal absorption of CUR in one lipidic nanosystem (i.e., self-microemulsifying systems) was passively transferred [38], greater efforts were still needed before we determine the transfer method of CUR in other lipidic nano-systems (i.e., CUR-C-HLN). Increased absorptive amount of CUR-C-HLN might be explained by the solubilization of CUR by solid and fluid liquidic matrixes; protection from elimination by incorporating CUR inside the nano-particles which contained surfactant inhibitor of enzyme CYP3A and protein P-gp [10, 24] (CUR was an enzymatic and proteinic substrate) [39]; a high dispersibility of CUR in binary lipids, surfactant-induced membrane fluidity; etc.

CUR-C-HLN displayed superior pharmacokinetic curves than free CUR after oral administration. The explanations for the maximal concentration peaks in the CUR-C-HLN curves are listed as follows: the first one was due to the presence of cationic, anionic and zwitter-ionic surfactants; the second one was because of a slow release of CUR incorporated in solid and fluid lipidic matrixes. Compared to free CUR, CUR-C-HLN had higher *in vivo* absorption, which was consistent with the outcome of *in situ* absorptive tests and *in vitro* release tests; CUR-C-HLN stayed longer time in plasma and cleared at a slower rate, which extended action time as well as effectiveness. The bioavailability of CUR-C-HLN to CUR was ~550%. Other systems (self-nano phospholipid dispersion or imprinted-like biopolymeric micelles) were documented to increase the CUR bioavailability whether or not they containing lipids [41]. After intravenous administration, the bioavailability of CUR solid lipidic nano-particles was lower as compared to that of CUR-C-HLN (125% versus 618%) [42].

CUR-C-HLN markedly enhanced the cell growth inhibition, apoptotic inducing and anti-invasion effects of CUR in lung adenocarcinoma cells. The IC_50_ value of CUR-C-HLN was only around one-fifth that of free CUR, indicating the much higher anticancerous effects of CUR-C-HLN. In recent decades, a few CUR delivery systems exhibited stronger cytotoxicities against cancerous cells compared to CUR. For example, PNIPAAm-MAA nanoparticles [43] inhibited the growth of breast cancer MCF-7 cells more efficiently, and PEG_2000_-oleoyl chloride diblock copolymer micelles suppressed proliferation of mammary and hepatocellular carcinoma 4T1 and HuH-7 cells more intensively [32]. Compared to free CUR, CUR-C-HLN induced a higher degree of S phase arrest and apoptosis. The induction of apoptosis might be involved with the decreased membranous potential and increased calcium-ion and active oxygen. CUR caused cell-cycle arrest with subsequent apoptosis in A549 cells [7, 44] and the apoptosis in NSCLC cells (H1299 and A549) in calcium-dependent ways [45]. CUR-C-HLN displayed better suppression of invasion compared to CUR. CUR inhibited cell growth and invasive ability in lung cancerous cells by modifying Wnt/β-catenin or Rac-dependent pathways [46, 47]. The anticancer activities of CUR were improved to various extent by most nano-carriers, and the action mechanisms varied according to the type of cancer [48].

Investigation on pharmacodynamics of CUR-C-HLN in cancerous mice showed that CUR-C-HLN had superior anti-lung tumor effects to that of free CUR. The reasons might be ascribed to the following characteristics: encapsulation of CUR inside C-HLN made it feasible for CUR to circulate in the blood system at relative high concentrations for much longer time, and then have much higher *in vivo* bioavailability; high-dispersity of CUR in hybrid lipids and catan-ionic surfactants were beneficial to increase the tumor uptake of CUR following endocytosis and membranous fusion [25]; CUR-C-HLN showed higher antitumor activity against cancerous cells via combinative mechanisms such as cell growth inhibition, apoptotic inducing and anti-invasion effects. Previously, it was reported that CUR suppressed lung cancer progression in C57BL/6 mice bearing xenografted cells via increase of the HIF1α/mTOR/VEGF/VEGFR cascade [49] and was effective in treating lung cancer by regulating oncogenes such as p53, enzymes such as LOX, transcription factors such as STAT-3 and protein kinases such as MAPK [50].

## MATERIALS AND METHODS

### Materials and animals

CUR was purchased from Sciphar Biotechnology Co. Ltd (Xi’an, China). Lipoid-S 75 was provided from Phospholipid GmbH. (Koeln, Germany). CTAB and SDS were purchased from Kelong Chemical Co. (China). Isopropyl palmitate was bought from Kingboc Co. (Jiaxing, China). The Sprague Dawley rats (230 ± 20 g) and C57BL/6J mice (20 ± 2 g) were supplied by the Laboratory Animal Center of Chongqing Medical University and Third Military Medical University (China). All animal experiments were performed in accordance with the protocol approved by the Laboratory Animal Committee of Medical Universities.

### Fabrication of CUR-C-HLN

CUR-C-HLNs were prepared by a thin film dispersion-sonication method. Glycerin monostearate, isopropyl palmitate, phospholipid and CUR were put in ethanol and subjected to sonication for 120 sec to obtain a yellowish solution. Then, the ethanol was evaporated at 30°C under hypobaric conditions until a light yellow film appeared. The film was then added to distilled water containing CTAB and SDS (1 mol: 2 mol) and sonicated for 10 min to obtain a yellow suspension. The optimal formula of CUR-C-HLNs was obtained by CCD-RSM method (Supplementary Table 2). The experimental results revealed that factors such as isopropyl palmitate and canta-ionic surfactant exerted strong influences on the encapsulate ratio and CUR content.

### Elementary characteristics of C-C-HLN

The features of CUR-C-HLNs in particulate diameter and charge were measured by Malven ZetaSizer (Nano-ZS90). The micromorphology was assessed by TEM method. The release amounts of CUR-C-HLNs in pH 1.2 or pH 6.8 aqueous media were measured by dialysis technique and calculated by model-fitting method [51]. The IR spectroscopic and calorimetric methods were applied to evaluate the entrapment of CUR in CUR-C-HLNs.

### Absorptive behavior in gastric and intestinal tracts

*In situ* gastro-intestinal perfusion methods [10, 28] were used to determine the absorptive behavior of CUR-C-HLN. In the gastric absorptive tests, the stomachs of anesthetized rats were perfused with CUR-C-HLNs, which remained there for 2 h before being drawn out and determined by HPLC. In the intestinal absorptive study, each duodenal, jejunal, ileal and colonic segment was attached to the perfusion assembly, which consisted of a BT100-1L peristaltic pump and equilibrated with Krebs-Ringer solution at 24 mL/h for a quarter. Intestinal segments were perfused with CUR-C-HLN at 12 mL/h for 1 h. The remaining perfusion was then determined by HPLC.

### Pharmacokinetic properties

CUR and CUR-C-HLN were administered to the rats via oral or intravenous routes. Blood taken from eyes were centrifuged and mixed with nitrendipine (0.1 mL) and ethyl acetate (1 mL). After centrifugation, the lower layer was dried and resolved with a mixed solution of acetonitrile and 5% acetic acid (55 mL:45 mL) for a HPLC analysis [7, 23]. The pharmacokinetic profiles were calculated by a DAS 2.1.1 software.

### Cell growth inhibition, apoptotic inducing and anti-invasion effects of CUR-C-HLN

The tumor cells were cultivated at a concentration of 5000 cells in each well. The media was substituted with different media containing different amounts of CUR-C-HLN in 2 d and 20 μL of MTT solution in another 2 d. The absorptive values were detected at 490 nm.

The tumor cells were added with CUR-C-HLN for 1 d and harvested by trypsinization. After centrifugation, they were fixed and then suspended in a PI/RNase solution for 0.5 h. The cellular cycle was evaluated by a FACSVantage flow cytometer [7]. The cellular apoptosis was investigated by an Annexin V-FITC-PI kit. The tumor cells were added with CUR-C-HLN for 1 d and stained with Rhodamine, Fluo 3 and DCFH for 0.5 h and determined using flowcytometer FACS Vantage.

The tumor cells added with CUR-C-HLN for 1 d were placed in the Matrigel-coated Transwell upper chamber and incubated for another 1 d. The invaded cells in the lower chamber were observed [52].

### Antitumor efficiency of CUR-C-HLN

C-HLN, 0.9% NaCl, CUR or CUR-C-HLN (50 μg per mouse) was given to tumor-bearing mice (C57BL-6J) one time every 48 h for 14 days via intraperitoneal route, respectively. Each group had 6 mice. The tumor volume and weight were recorded 48 h later after the last administration [53, 54].

## CONCLUSIONS

A new kind of catan-ionic hybrid lipidic nano-carriers (C-HLN) was devised to deliver CUR and overcome its defects. Compared to CUR, CUR-C-HLN improved absorptive and pharmacokinetic behaviors, increased the tumor cell growth inhibition, apoptotic inducing and anti-invasion effects, enhanced the antitumor efficiency. This work was the first to report on fabricating catan-ionic hybrid lipidic nano-particles to deliver insoluble antitumor drugs to treat tumor. The CUR-C-HLN provides a useful option for efficient delivery of anti-tumor chemodrugs.

## SUPPLEMENTARY INFORMATION

Supporting Information is available free of charge on the website at http://dx.doi.org/.

## SUPPLEMENTARY MATERIALS TABLES


