# Supplementary material for: Catan-ionic hybrid lipidic nano-carriers for enhanced bioavailability and anti-tumor efficacy of chemodrugs
Source: Oncotarget. 2017 Mar 6;8(19):30922–32. doi: 10.18632/oncotarget.15942 (PMC5458178; doi:10.18632/oncotarget.15942)
Supplement: Supplementary file 1 [file oncotarget-08-30922-s001.pdf]

Catan-ionic hybrid lipidic nano-carriers for enhanced bioavailability and anti-tumor efficacy of chemodrugs

Supplementary Materials

Supplementary Table 1: The pH and electrical conductivities of CUR-C-HLN and free CUR

Parameter	Water	Free CUR in water	Blank C-HLN	CUR-C-HLN
pH	6.78 ± 0.02	7.22 ± 0.02	5.84 ± 0.03	5.83 ± 0.01
Conductivity (μs/cm)	15.73 ± 0.01	18.18 ± 0.02	616.00 ± 2.00	613.33 ± 3.21

Supplementary Table 2: Composition of central composite design batches (mean ± SD, *n* = 3)

Batches	X_1	X_2	X_3	X_4	Y_1 (Entrapment efficiency, %)	Y_2 (Drug loading, %)
1	70.24	32.14	70.24	105.00	41.97 ± 2.26	2.21 ± 0.31
2	129.76	32.14	70.24	105.00	12.03 ± 1.30	3.01 ± 0.15
3	100.00	50.00	100.00	150.00	89.03 ± 1.33	4.67 ± 0.40
4	129.76	67.86	70.24	195.00	38.69 ± 4.49	1.42 ± 0.43
5	100.00	20.00	100.00	150.00	29.58 ± 4.38	3.89 ± 0.31
6	129.76	67.86	129.76	195.00	45.92 ± 2.39	3.03 ± 0.21
7	129.76	67.86	70.24	105.00	40.13 ± 2.11	1.08 ± 0.23
8	70.24	67.86	129.76	105.00	79.15 ± 1.76	5.03 ± 0.63
9	100.00	50.00	100.00	150.00	89.08 ± 1.00	4.89 ± 0.15
10	129.76	32.14	129.76	105.00	48.51 ± 3.29	3.13 ± 0.34
11	100.00	50.00	100.00	74.40	62.87 ± 2.02	2.49 ± 0.02
12	129.76	67.86	129.76	105.00	51.06 ± 3.78	2.90 ± 0.45
13	100.00	80.00	100.00	150.00	61.16 ± 2.53	3.17 ± 0.30
14	70.30	67.86	70.24	105.00	85.42 ± 1.71	2.07 ± 0.23
15	70.24	32.14	129.76	105.00	55.39 ± 5.06	4.13 ± 0.21
16	70.24	32.14	129.76	195.00	57.00 ± 2.92	6.75 ± 0.33
17	70.24	67.86	129.76	195.00	70.94 ± 4.11	6.48 ± 0.55
18	70.24	67.86	70.24	195.00	85.72 ± 5.85	4.54 ± 0.36
19	129.76	32.14	70.24	195.00	35.97 ± 4.26	5.15 ± 0.23
20	100.00	50.00	100.00	150.00	89.25 ± 3.91	5.12 ± 0.12
21	50.00	50.00	100.00	150.00	96.29 ± 2.14	5.98 ± 0.53
22	100.00	50.00	100.00	225.60	70.37 ± 2.04	5.65 ± 0.31
23	129.76	32.14	129.76	195.00	63.86 ± 4.93	3.27 ± 0.20
24	150.08	50.00	100.00	150.00	58.77 ± 4.63	3.78 ± 0.39
25	100.00	50.00	100.00	150.00	85.78 ± 3.21	4.89 ± 0.17
26	100.00	50.00	50.00	150.00	88.73 ± 0.64	2.29 ± 0.10
27	100.00	50.00	100.00	150.00	85.79 ± 2.08	5.74 ± 0.33
28	100.00	50.00	100.00	150.00	85.98 ± 3.19	4.98 ± 0.54
29	100.00	50.00	150.00	150.00	84.98 ± 2.79	5.91 ± 0.36
30	70.24	32.14	70.24	195.00	58.30 ± 6.65	6.20 ± 0.09

Supplementary Table 3: Comparison of observed and predicted values of CUR-C-HLN prepared under the optimal protocol (mean ± SD, n = 3)

Formulation characteristics	Predicted value	Observed value	Bias* (%)
Entrapment efficiency (%)	93.23	94.38 ± 2.67	1.23
Drug loading capacity (%)	6.75	6.93 ± 0.39	2.62

*Bias was calculated according to this formula: Bias (%) = (Predicted value - Observed value)/Predicted value ×100%.

Supplementary Table 4: The similarity between the release profiles of CUR-C-HLN and CUR

Release profile 1		Release profile 2		f_2	Difference
Formulation	Release medium	Formulation	Release medium		
CUR-C-HLN	pH 1.2 HCl solution	CUR	pH 1.2 HCl solution	47.84	+
CUR-C-HLN	pH 6.8 PBS	CUR	pH 6.8 PBS	45.74	+
